# Characteristics of vasculogenic mimicry and tumour to endothelial transdifferentiation in human glioblastoma: a systematic review

**DOI:** 10.1186/s12885-023-10659-y

**Published:** 2023-02-23

**Authors:** Kelsey Maddison, Nikola A. Bowden, Moira C. Graves, Paul A. Tooney

**Affiliations:** 1 Medical Sciences Building, School of Biomedical Sciences and Pharmacy, The University of Newcastle, University Drive, 2308 Callaghan, NSW Australia; 2 School of Medicine and Public Health, The University of Newcastle, Callaghan, NSW Australia; 3 Mark Hughes Foundation Centre for Brain Cancer Research, The University of Newcastle, Callaghan, NSW Australia; 4 Drug Repurposing and Medicines Research Program, Hunter Medical Research Institute, New Lambton Heights, NSW Australia

**Keywords:** Glioblastoma, Vasculogenic mimicry, Transdifferentiation, Tumour-derived vasculature

## Abstract

**Background:**

Glioblastoma, the most common primary malignant brain tumour in adults, is a highly vascular tumour characterised by abnormal angiogenesis. Additional mechanisms of tumour vascularisation have also been reported in glioblastoma, including the formation of tumour cell-derived vessels by vasculogenic mimicry (VM) or the transdifferentiation of tumour cells to endothelial cells. VM and endothelial transdifferentiation have frequently been reported as distinct processes, however, the use of both terms to describe a single process of vascularisation also occurs. Some overlapping characteristics have also been reported when identifying each process. We therefore aimed to determine the markers consistently attributed to VM and endothelial transdifferentiation in the glioblastoma literature.

**Methods:**

Ovid MEDLINE and Ovid Embase were searched for studies published between January 1999 and July 2021 that assessed VM or tumour to endothelial transdifferentiation in human glioblastoma. The online systematic review tool Covidence was used for screening and data extraction. Extracted data included type of tumour-derived vasculature reported, methods and techniques used, and markers investigated. Studies were grouped based on type of vasculature reported for further assessment.

**Results:**

One hundred and thirteen of the 419 unique records identified were included for analysis. VM was reported in 64/113 studies, while tumour to endothelial transdifferentiation was reported in 16/113 studies. The remaining studies used both terms to describe a single process, did not define the process that occurred, or concluded that neither VM nor endothelial transdifferentiation occurred. Absence of CD34 and/or CD31 in vascular structures was the most common indicator of VM, while expression of CD34 and/or CD31, in addition to various other endothelial, stem cell or tumour cell markers, indicated tumour to endothelial transdifferentiation.

**Conclusion:**

Cells derived from tumour to endothelial transdifferentiation express typical endothelial markers including CD34 and CD31, while tumour cells contributing to VM lack CD34 and CD31 expression. Additional tumour markers are required to identify transdifferentiation in glioblastoma tissue, and this process requires further characterisation.

**Supplementary Information:**

The online version contains supplementary material available at 10.1186/s12885-023-10659-y.

## Background

Angiogenesis is a complex, highly regulated process requiring the interaction of various cell types, including endothelial cells and pericytes, extracellular matrix components, proteolytic enzymes, and soluble factors for the successful growth of mature, stable, and functional vascular networks [1]. Tumour angiogenesis involves persistent pro-angiogenic signalling, and reduction in anti-angiogenic signalling, and results in poorly formed, dysfunctional vasculature. In addition to causing excessive endothelial proliferation and vessel branching, the imbalance in angiogenic signalling in tumour angiogenesis leads to loose association with, or lack of coverage by, pericytes and therefore vessel instability [2]. It is now recognised that tumours may acquire their blood supply not only through angiogenesis but also through alternative vascularisation processes, including the contribution of tumour cells themselves to vessel development. Two of these tumour cell-derived vascularisation processes, vasculogenic mimicry and tumour to endothelial cell transdifferentiation, are the focus of this review.

The term “vasculogenic mimicry” (VM) was first used by Maniotis et al. in 1999 to describe the patterns of functional microcirculation generated by tumour cells and lacking an endothelial lining observed in uveal and metastatic cutaneous melanoma tissue [3]. Vascular structures in this initial report were described as loops and networks that showed positive staining with periodic acid-Schiff (PAS) and may have solid and/or hollow components, with the hollow channels sometimes containing red blood cells. Endothelial cells were not identified in these vessel-like channels by microscopy or immunohistochemical labelling for various endothelial markers, including CD31 and CD34 [3]. Presence of PAS-positive (PAS+) networks was consistent with more aggressive and invasive melanomas and was associated with death from metastatic disease in uveal melanoma. Melanoma cell lines with the invasive phenotype were also able to form similar networks to those seen in tissue when grown in vitro on Matrigel® or Type I collagen, while less invasive melanoma cells and normal melanocytes could not.

The VM networks formed by melanoma cells have been described as resembling the networks formed during embryonic vasculogenesis, the de novo formation of a primitive vascular network by endothelial precursor cells in the developing embryo [4]. Research in melanoma has identified several markers associated with both vascular and embryonic signalling pathways in VM-capable cells [5–9]. Plasticity is frequently described as an important characteristic for VM, and embryonic and stem cell signalling are involved in its formation through Notch and Nodal pathways [8]. Several markers of VM, such as vascular endothelial (VE)-cadherin [5, 10], EphA2 [10, 11], matrix metalloproteinase (MMP)-2 [7, 12], membrane type 1 (MT1)-MMP (MMP-14) [7, 12], and the basement membrane component laminin 5 [7, 12], are also associated with vasculogenesis and/or angiogenesis. Lack of the endothelial marker CD31 has also been reported subsequent to the initial VM study [5]. Additionally, signalling pathways associated with hypoxia are associated with VM formation [13, 14], suggesting that in addition to promoting angiogenesis, the hypoxic tumour microenvironment may promote VM.

Vasculogenic mimicry, also referred to as vascular mimicry, has now been reported in several other tumour types, including glioblastoma [15–17]. Glioblastoma is a CNS WHO grade 4 diffuse glioma and is the most common primary malignant brain tumour in adults [18, 19]. Glioblastomas are aggressive, treatment-resistant tumours and have poor prognosis and survival outcomes, with a median survival time of only 14.6 months with the current standard therapy [20]. Diagnosis of glioblastoma includes both histological and molecular criteria, with the presence of wildtype isocitrate dehydrogenase (*IDH*) gene(s) being necessary [18]. Glioblastomas are both hypoxic and highly vascular, with abnormal angiogenic activity in the form of microvascular proliferation being a typical histological feature [21]. The anti-vascular endothelial growth factor (VEGF) antibody bevacizumab may be used in recurrent tumours to target angiogenesis [22–24]. However, bevacizumab treatment has not demonstrated overall survival benefit in either primary or recurrent glioblastoma and treatment resistance often occurs [23–25]. Formation of vasculature through alternate mechanisms, such as VM, may contribute to treatment resistance to anti-angiogenic therapies in glioblastoma.

Additional forms of tumour cell-derived vasculature have also been identified in glioblastoma, including the transdifferentiation of cancer stem-like cells into functional endothelial cells that participate in vessel formation [26]. Researchers have identified a subset of endothelial cells in glioblastoma tissue that display the same genetic aberrations as surrounding tumour cells and thus propose that these cells are derived from tumour cells [26, 27]. A fraction of stem-like cells isolated from glioblastomas have been shown to express a number of endothelial cell markers including CD31, CD34, CD105, VE-cadherin, von Willebrand factor (vWF), and VEGF receptor 2 (VEGFR2) [26, 28, 29]. When cultured in endothelial growth medium and grown on Matrigel®, these cells are also able to form tubes in vitro, resembling vessels formed by normal endothelial cells [26, 27, 29].

While VM and tumour to endothelial transdifferentiation are frequently reported as individual processes, an overlap in terminology has also started to occur in the glioblastoma literature. For example, some studies indicate that VM occurs as the result of endothelial transdifferentiation of tumour cells. Additionally, expression of some markers, such as VE-cadherin, or the impact of microenvironmental conditions, such as hypoxia, are reported as contributing to both processes. This has resulted in some confusion relating to the understanding of the characteristics that distinguish these forms of tumour-derived vasculature. Therefore, this systematic review aimed to assess the descriptions in the literature attributed to vasculogenic mimicry and endothelial transdifferentiation in relation to human glioblastoma, and to determine the characteristics and techniques that are used to identify each vascularisation process.

## Methods

### Protocol registration

As this review deals with preclinical studies involving in vitro experiments, there is currently no suitable repository in which the protocol for this review could be registered. Preferred Reporting Items for Systematic Reviews and Meta-Analyses (PRISMA) guidelines were followed to the extent that this was applicable.

### Eligibility criteria

Studies that assessed vasculogenic mimicry and/or tumour cell to endothelial cell transdifferentiation in human glioblastoma cells, cell lines, or tissues were considered eligible for inclusion, regardless of study design. This included in vitro and in vivo studies. Only original research studies were included; review articles and conference abstracts were excluded. Studies in which all experiments related to any of the following criteria were excluded: paediatric glioblastoma, brain tumour types other than glioblastoma, assessment of only animal cells, animal cell lines or animal tissues, differentiation or transdifferentiation of tumour cells into cell types other than endothelial cells, assessment of vascularisation processes that were not tumour cell-derived (e.g. angiogenesis).

### Information sources & search strategy

The electronic databases Ovid MEDLINE and Ovid Embase were searched on 8th July 2021 using various search terms relating to the concepts of glioblastoma and tumour-derived vasculature, specifically vasculogenic mimicry and/or the transdifferentiation of tumour cells to endothelial cells. Searches were limited to studies conducted from January 1999 to July 2021, as 1999 was the year in which the term “vasculogenic mimicry” was first attributed to tumour cell-lined vascular channels in melanoma [3]. The complete search strings used for each database are included in Supplementary Tables 1 & 2.

### Study selection

All records retrieved from the database searches were imported into the online systematic review tool Covidence for screening and data extraction. Records were automatically de-duplicated upon importing into Covidence, and any duplicate references that were not detected by this process were manually identified and removed. Title and abstract screening and full text screening were performed independently by two reviewers (KM and PAT), and discrepancies between reviewers were resolved by discussion. Studies that were published in languages other than English were excluded at the full text review stage due to inability to translate these studies. This allowed for identification and reporting of the number of studies that were potentially relevant but were excluded on the basis of language. We acknowledge that this introduced a language bias into the study selection process.

### Data extraction

Data extraction was performed independently by two reviewers (KM and PAT) using a data extraction form created in Covidence. The extracted data included the following:

- General information: title, authors, year of publication, and study aim.
- Type(s) of tumour-derived vasculature investigated, including terminology used, definitions provided, and type(s) of vasculature reported in the study based on cellular/tissue characteristics that were observed.
- Whether the study used tissue and/or in vitro and/or in vivo techniques, and, where relevant, details relating to the techniques used and markers assessed:
  - tissue studies: type of glioblastoma tissue (primary or recurrent), techniques used, markers assessed.
  - in vitro studies: type and names of cells/cell lines used, control cells, techniques used, markers assessed, timepoint(s) at which observations were made, growth conditions, whether a tube formation assay was performed, whether vessel functionality was assessed.
  - in vivo studies: type of cells/cell lines used, techniques used, markers assessed, whether vessel functionality was assessed.

### Quality assessment

As we were interested in further understanding the variation and overlap in descriptions of vasculogenic mimicry and endothelial transdifferentiation in glioblastoma, including variation in methods and results on which conclusions on vasculature were based, we have not applied a quality assessment step. Additionally, no pre-existing, standardised risk of bias or quality assessment tool was identified as being suitable for use with all types of studies included in this review.

### Data synthesis

The proportion of studies describing VM, tumour to endothelial transdifferentiation, both processes, or neither process was reported. Studies were divided into groups based on type of tumour-derived vasculature that was described. Within the VM and tumour to endothelial transdifferentiation groups, the most common techniques used to assess vasculature, and markers or characteristics attributed to each type of vasculature were determined as a proportion of studies included in each subgroup.

## Results

### Study selection and characteristics

**Fig. 1:**
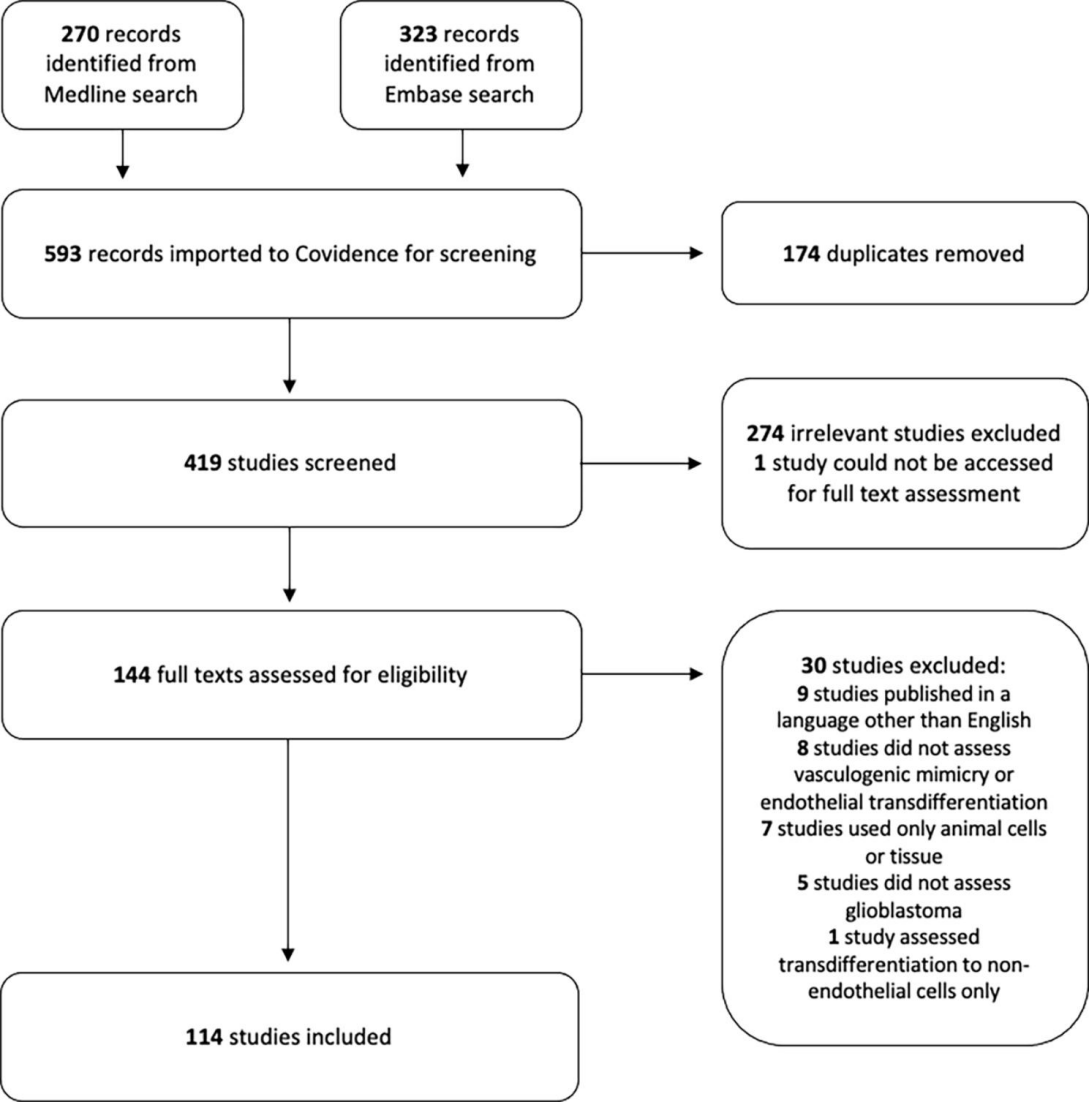
Study selection flow diagram

The database searches identified a total of 593 records from Medline and Embase, all of which were retrieved. Following deduplication, 419 records underwent title and abstract screening, and 144 potentially relevant full texts were assessed for eligibility. Of these studies, 114 were included for analysis. The study selection process and exclusion reasons are detailed in Fig. 1. During the preparation of this manuscript, one included study was retracted and has been excluded from the below results, which are therefore described for a total of 113 studies.

The year of publication for the included studies ranged from 2005 to 2021. Of the 113 studies identified as assessing VM and/or endothelial transdifferentiation in glioblastoma, 54 studies (47.79%) analysed human tissue samples, 94 studies (83.19%) used in vitro methods, and 59 studies (52.21%) used in vivo methods.

### Definitions of tumour-derived vasculature in glioblastoma

VM was more frequently described than tumour to endothelial transdifferentiation, with 60/113 studies (53.1%) mentioning or defining VM as a distinct process compared to 16/113 (14.2%) that described tumour to endothelial differentiation or transdifferentiation. Twelve studies (10.6%) mentioned both terms and considered them separate processes, while nine studies (8.0%) used both terms to describe a single form of tumour-derived vasculature. Sixteen of the 113 studies (14.2%) did not mention or provide any description of tumour-derived vasculature prior to presenting or discussing experimental results. Seven studies (6.2%) also provided descriptions of tumour-derived vascularisation processes that were not either VM or endothelial transdifferentiation, e.g. tumour to pericyte transdifferentiation, in addition to one or both of the processes of interest. Subgroups of studies that provided a clear definition and assessment of either VM (64/113) or tumour to endothelial cell (trans)differentiation (16/113) were further evaluated to determine the methods and markers used to identify each type of tumour-derived vasculature.

### Vasculogenic mimicry in glioblastoma

Of the 60 studies that mentioned VM only in their description of tumour-derived vasculature, all suggested that their results indicated that the type of vessels observed were VM. Additionally, four of the 12 studies that mentioned both VM and endothelial transdifferentiation as separate processes concluded that the type of vasculature observed was VM, and these studies were therefore included when determining markers used to identify VM.

In the 64 studies that assessed VM, in vitro methods were most commonly used (55/64), followed by tumour tissue (35/64), then in vivo methods (32/64). The most frequently used techniques in in vitro studies were tube formation assay (TFA; 49/55), Western blot (34/55), PCR (22/55), immunofluorescence (IF) (8/55), and ELISA (6/55) (Supplementary Table 5). All studies that used TFAs assessed the ability of tumour cells to undergo VM based on morphological changes and reported varying descriptions of VM formation, for example, tube-like structures, capillary-like structures, cavity structures, branching, loops, or networks. Several aspects of the TFA varied between studies, such as use of commercial or patient-derived cells, growth conditions, and incubation time. The majority of glioblastoma cells used for TFAs were commercially available cell lines (e.g. U87, U251, A172), which were used in 46/49 studies, compared to five studies using patient-derived cells. Fourteen studies used endothelial cells (10/49 used HUVECs, 4/49 used HMVECs) as positive controls to evaluate the VM capability of glioblastoma cells, whilst 32 studies did not compare tube formation to a control cell line.

Though hypoxia-related signalling has previously been associated with the formation of VM, only six studies compared tube formation under both hypoxic and normoxic conditions. All six studies determined that tube formation was induced or enhanced by hypoxia in VM-capable cells, over periods of time ranging from 2 to 24 h. Time from cell plating to image capture/analysis ranged from two hours to six days, with 11/49 studies collecting image data at multiple timepoints. No study that performed a TFA to assess VM conducted functional assessment of the resulting vessel-like structures. One study used IF in combination with the TFA and observed that VEGFR2, laminin 5 γ2 chain, and the mural cell marker smooth muscle actin (SMa) were expressed by glioblastoma cells that were capable of VM, and that these cells lacked the endothelial cell markers CD31, VE-cadherin, Tie1, and Tie2 [30]. Three studies that assessed CD31 expression by Western blot or PCR agreed that VM-capable glioblastoma cells lacked CD31. VE-cadherin was one of the most evaluated markers by Western blot, however, conclusions regarding the involvement of VE-cadherin in VM were inconsistent, with eight studies reporting an association between VE-cadherin expression and the ability of cells to form tube-like structures in vitro, and another four studies reporting that VE-cadherin was not expressed by VM-capable cells or that its expression was not associated with tube formation ability. Other commonly evaluated markers were some of those that have previously been reported as VM-associated in melanoma, including MMP-2, MMP-9, and EphA2 (Supplementary Table 6). Associations were reported between VM and MMP-2 expression by 15 studies (7 by Western blot, 2 by PCR, 6 used both), VM and MMP-9 expression by 11 studies (7 by Western blot, 2 by PCR, 4 used both), and VM and EphA2 expression by five studies (all PCR).

All 35 studies that assessed glioblastoma tissue performed immunohistochemistry (IHC). Forty-two unique IHC markers were assessed across the 35 studies (Supplementary Table 4). PAS staining to visualise vascular basement membranes was the most frequently used marker (34/35) and was used in combination with CD34 (28/35) and/or CD31 (8/35) to identify VM vessels. VM vessels were defined as CD34-/PAS+ structures by 24/28 studies using CD34, while the remaining four studies did not provide a description of how the markers were used to identify VM vessels. All studies that used CD31 as an endothelial cell marker identified VM structures as being CD31-/PAS+. Fourteen studies also noted that red blood cells were observed within VM vessels. None of these studies mentioned whether white blood cells were also present. Five studies labelled tissue sections with glial fibrillary acidic protein (GFAP) to identify tumour cells. Of these studies, three used GFAP labelling to confirm that cells lining VM vessels were tumour cells. Four studies labelled for the pericyte marker aSMa, two of which concluded that VM structures are composed of mural-like tumour cells. Other techniques used were IF (6/35), fluorescence in situ hybridisation (FISH) (2/35), PCR (2/35), and Western blot (1/35) (Supplementary Tables 3 & 4). Five of 35 studies specified that glioblastoma tissue was collected from primary tumours; the remaining 30 studies did not specify whether primary or recurrent tissue was assessed.

All studies that assessed VM in in vivo models also used IHC. As with the studies that assessed human tissue, PAS staining was used in the majority of studies (30/32) in combination with CD34 (18/32) and/or CD31 (9/32). Antibodies directed against both human and mouse CD31 and/or CD34 were occasionally used to determine the presence of VM. In three studies that used human CD31 (hCD31) and mouse CD31 (mCD31) antibodies, two studies reported that PAS+ vessels that lacked both hCD31 and mCD31 indicated VM, while the remaining study defined VM vessels as hCD31+/PAS+ [31]. Other IHC markers assessed by more than one study each were GFAP (3/32), SMa (2/32), and Tenascin-C (2/32). IF was used by five studies to assess in vivo VM, which was identified using various combinations of markers, including GFAP+/CD34- and SMa+/GFAP+/VEGFR2+/hCD31-/hCD34-. The same study that reported hCD31+/PAS+ vessels by IHC also defined VM vessels as hCD31+ by IF[31]. Additionally, one study reported localisation of VE-cadherin and EphA2 to vessels, but did not confirm by IF that cells lining the vessel were tumour cells rather than endothelial cells [32]. Techniques and markers used to assess VM in vivo are summarised in Supplementary Tables 7 & 8. As with the in vitro assessments of VM, in vivo models more commonly used commercial cell lines (29/32) than patient-derived tumour cells (6/32). Five studies reported that VM vessels were functional based on presence of red blood cells in vessel lumen (2/5), injection of Evan’s blue dye (2/5), or staining of lectin perfused vessels that were lined or partially lined by tumour cells (1/5).

### Tumour to endothelial transdifferentiation

Of the 16 studies that described tumour-derived vessels forming by transdifferentiation of tumour cells into endothelial cells, 12 studies concluded that tumour to endothelial transdifferentiation was observed. From the group of studies that defined VM and endothelial transdifferentiation as separate processes, four studies concluded that transdifferentiation was observed, and have been included in the group of studies (n = 16) used to evaluate the characteristics of endothelial transdifferentiation in glioblastoma.

The ability of glioblastoma cells to transdifferentiate into endothelial cells was most frequently assessed using in vitro techniques (13/16 studies). TFAs were again one of the most commonly used techniques (9/13), with morphological changes indicating endothelial phenotype. One study used IF to label tumour-derived endothelial cells that had undergone tube formation and showed that they expressed CD105 (endoglin) [26], a cell surface glycoprotein normally expressed by vascular endothelial cells that is associated with endothelial proliferation and angiogenesis[33]. Another study harvested glioblastoma cells after they had undergone tube formation and performed Western blots to determine their expression of the endothelial markers CD31 and ETS transcription factor ERG (ERG) [34]. Both ERG and CD31 were expressed by glioblastoma stem-like cells capable of tube formation, indicating transdifferentiation to endothelial cells. In contrast to studies assessing VM, patient-derived cells were more commonly used (7/9) than commercial cell lines (5/9), and all studies that performed a TFA to identify endothelial transdifferentiation used glioblastoma stem-like cells (GSCs) isolated either from patient-derived or commercial cell lines. Five of nine studies did not compare tube formation by glioblastoma-derived endothelial cells to a control cell line (5/9), while the remaining four studies compared tube formation to a normal endothelial cell line (2/9 used HUVECs, 1/9 used human brain endothelial cells, and 1/9 used human dermal microvascular endothelial cells). Tube formation was usually assessed under normoxic conditions only (5/9), with only one study assessing under hypoxic conditions [29]. One study compared normoxic and hypoxic conditions and reported increased tube formation with hypoxia [28]. The remaining two TFA studies did not specify whether cells were grown in normoxic or hypoxic conditions. Time from cell plating to image capture and/or analysis ranged from 15 min to 8 days, with 4/9 transdifferentiation studies assessing tube formation across multiple timepoints. In contrast to VM, where only one study assessed tube formation using endothelial cell growth medium (EGM) [16], GSCs in transdifferentiation studies were often plated in an EGM for the duration of the TFA (4/9), and/or grown in EGM to promote transdifferentiation prior to undergoing the TFA (5/9). Two studies determined that transdifferentiated glioblastoma cells displayed functional characteristics of endothelial cells using fluorescently labelled acetylated low-density lipoprotein (DiI-Ac-LDL) uptake assays.

Endothelial transdifferentiation was also assessed by PCR in 9/13 in vitro studies, most frequently using the markers CD31 (6/9), VE-cadherin (4/9), CD34, VEGFR2, and vWF (each 3/9). One study compared VEGFR2 expression between GSCs, differentiated glioblastoma cells, and endothelial cells derived from GSCs and did not identify a significant difference in VEGFR2 expression between these cell types [35]. All other studies assessing CD31, VE-cadherin, CD34, VEGFR2, and vWF determined that mRNA expression of these markers was associated with the transdifferentiation of glioblastoma cells to endothelial cells. CD31 and CD34 were also identified as being associated with endothelial transdifferentiation and/or acquisition of endothelial phenotype by additional methods (Supplementary Tables 11 & 12) including IF, where six studies assessed CD31 and four studies assessed CD34.

Endothelial transdifferentiation was assessed using in vivo models in 12/16 studies, with IF and IHC used by six studies each. The most common method of identifying glioblastoma-derived endothelial cells in IF studies was through the use of both human- and mouse-specific anti-CD31 antibody labelling (3/6). In these studies, vessels containing glioblastoma-derived endothelial cells were identified by their expression of human-specific CD31. In IHC studies, human-specific CD31 was the most frequently assessed marker, used by 2/6 studies. All other markers assessed were used by one study each (Supplementary Tables 13 & 14). Patient-derived cells were more frequently used (9/12) to study endothelial transdifferentiation in vivo than commercial cell lines (3/12). Three studies noted that vessels formed by glioblastoma-derived endothelial cells were functional, each using a different method to assess this (presence of red blood cells in vessel lumen in H&E sections, uptake of systemically injected lectin, and presence of systemically injected absorbate particles within the vessels).

Seven studies assessed endothelial transdifferentiation using human glioblastoma tissue. One study assessed primary tumours only, two studies assessed both primary and recurrent tumours, and the remaining four studies did not specify whether tumours were primary or recurrent. Five studies used IF to demonstrate expression of various endothelial and stem cell markers, most commonly CD31 (3/7), CD34, vWF, and Nestin (each 2/7). Two studies used IF in combination with FISH to determine whether cells that lined blood vessels and expressed endothelial markers also demonstrated amplification of epidermal growth factor receptor (EGFR), a common genetic alteration in glioblastoma [36]. Both studies observed EGFR amplification in a proportion of endothelial-like cells and suggested that these cells were of glioblastoma origin. Four studies used IHC, with Nestin being the only marker assessed by more than one study (2/4). All techniques and markers used to assess tumour to endothelial transdifferentiation in human tissue are summarised in Supplementary Tables 9 & 10.

## Discussion

This systematic review identified 113 papers that used terms relating to either or both of the concepts of vasculogenic mimicry and endothelial transdifferentiation in reference to tumour blood vessel formation in glioblastoma. Although VM and vessel formation by tumour-derived endothelial cells have frequently been described as separate vascularisation processes, nine papers used both terms/concepts to describe a single form of tumour-derived vasculature. While some studies suggested that VM may be an earlier stage of the transdifferentiation process, or vice versa, a number of descriptions using terminology referring to both concepts were not clear about whether VM, endothelial transdifferentiation, or a single multi-stage process was being investigated. We identified 64 studies that reported cellular and tissue markers and phenotypes specifically in relation to VM, and 16 studies that did the same for tumour to endothelial transdifferentiation. We used these subsets of studies to assess the characteristics that distinguish VM from endothelial transdifferentiation.

VM in glioblastoma tissue is described similarly to early reports of VM in melanoma; PAS+ basement membrane-lined structures without an endothelial cell lining, sometimes containing red blood cells [3]. Lack of an endothelial cell lining is confirmed by IHC using labelling for CD31 or CD34 in VM studies across various cancer types [37–41]. While both CD31 and CD34 labelling may be used in combination with PAS staining to distinguish VM vessels (PAS+/CD31- or PAS+/CD34-) from endothelial vessels (PAS+/CD31+ or PAS+/CD34+), the majority of studies that assessed VM in glioblastoma using IHC confirmed the lack of endothelial lining by labelling tissue for CD34. Two studies compared labelling of CD31 and CD34 for identification of endothelial vessels in astrocytoma tissue, with one study finding that CD34 provided more distinct labelling, and the other noting similar results with both markers [15, 42]. The observation of red blood cells within VM structures suggests that they make a functional contribution to the tumour vasculature. A relationship between lower endothelial vessel density and the presence of VM has also been reported in glioma[17], indicating that VM complements angiogenic vessel growth and may compensate for lack of blood supply to tumour tissue when angiogenesis is insufficient or disrupted by anti-angiogenic treatment. The collective results of the studies included in this review suggest that identification of VM in glioblastoma tissue can be achieved by IHC using either CD31 or CD34 labelling in combination with PAS staining of the basement membrane of the vessel structure.

Identification of glioblastoma-derived endothelial cells in tissue samples also commonly used CD31 and CD34 as markers. In contrast to VM, presence of CD31 or CD34 in cells that co-expressed tumour markers (e.g. EGFR amplification [26, 43]) or stem cell markers (e.g. CD133 [44], Nestin[44, 45]) indicated vessels containing endothelial cells resulting from transdifferentiation of GSCs. However, no tumour or stem cell marker was consistently used across multiple studies to identify tumour to endothelial transdifferentiation, though Nestin was used slightly more frequently than other markers. Both Nestin, an intermediate filament protein, and CD133, a stem cell marker frequently used to isolate cancer stem cells, are expressed by normal neural stem and progenitor cells and have been previously used to identify and isolate glioblastoma stem-like cells [46–49]. Accumulation of Nestin+ or CD133+ cells around glioblastoma vasculature was reported by one study that performed IHC and IF for these markers to assess transdifferentiation of GSCs to endothelial cells [44]. This pattern of labelling may however indicate colocalisation of cancer stem cells with capillaries, as has previously been reported in glioblastoma and other brain tumours [46], rather than co-expression of endothelial and stem cell markers. Chromosomal aberrations in CD31-expressing cells have also been suggested to indicate tumour to endothelial transdifferentiation in glioblastoma [26, 27]. However, chromosomal abnormalities, such as aneuploidy, have been reported in endothelial cells isolated from tumour tissue[50, 51]. A study that isolated murine CD31+ endothelial cells from human melanoma and liposarcoma xenografts demonstrated that a subset of these endothelial cells were aneuploid [51]. Normal endothelial cells were also isolated from non-tumour tissue for comparison, and these cells were diploid when freshly isolated and remained diploid after multiple passages in vitro [51]. A subsequent study demonstrated induction of aneuploidy in endothelial cells exposed to hypoxic conditions in vitro and in vivo, suggesting that chromosomal aberrations in endothelial cells may result from tumour microenvironmental factors [52]. While tumour to endothelial transdifferentiation may still represent a mechanism by which endothelial cells develop genetic alterations, relatively few studies have been conducted to identify tumour to endothelial transdifferentiation in glioblastoma tissue. Based on the current literature, tumour to endothelial transdifferentiation can be identified by the expression of CD31 and/or CD34 in tumour cells, as absence of these markers is consistently reported in VM. Ideally, endothelial marker expression would be assessed in combination with a glioblastoma-specific marker to confirm the tumour origin of vessel-lining cells. However, further characterisation of potential tumour-derived endothelial cells needs to occur before firm conclusions can be drawn regarding the extent of their presence in glioblastoma tissue and the optimal markers for their identification.

VM in xenograft tissue was again described as PAS+ and negative for endothelial markers CD34 and/or CD31. Interestingly, studies that used both serum-cultured commercial cell lines and GSC-only populations to generate xenograft tumours reported these VM structures. GSCs have been reported to more accurately recapitulate the genetic and phenotypic features of human glioblastoma than serum-cultured cell lines, with xenograft models developed using glioma stem cells resulting in highly vascular tumours with histological features such as palisading necrosis, microvascular proliferation, and cellular heterogeneity, and demonstrating infiltration into surrounding brain tissue [53, 54]. In comparison, some models using serum-cultured cell lines lack the intratumoural heterogeneity and diffuse infiltration seen in patient tumours [53, 55]. However, cell lines such as U87 and U251, both of which were used by studies included in this review, are reported to contain CD133+ stem-like cell populations [56]. This may contribute to their capacity to form VM structures, as embryonic and stem cell signalling pathways have previously been associated with VM [8].

Xenograft tumours formed by implantation of GSC-only populations were also reported to contain tumour-derived endothelial cells [26, 29, 34, 35]. Identification of endothelial transdifferentiation in xenograft tissue was frequently based on CD31 expression, with tumour-derived endothelial cells possibly being more easily distinguished in xenograft tissue than human tissue. Rather than the combination of endothelial and tumour cell markers that indicated endothelial transdifferentiation in human tissue, reactivity to human-specific antibodies indicated that cells expressing endothelial markers or vessel morphology were derived from tumour cells. The use of xenograft models also allows for the assessment of both tumour to endothelial transdifferentiation and VM within the same tissue sections. For example, Porcù et al. [29] observed both tumour-derived endothelial cells, based on human-specific CD31 labelling, and PAS+ VM structures that were negative for human- and mouse-specific CD31 in their orthotopic xenografts. Evaluating the expression of additional markers reported in these tumour vascularisation process in vivo could provide further insight into their differing, or shared, features.

The ability of tumour cells to undergo VM in vitro was also initially reported in melanoma cells [3]. A protocol for the in vitro assessment of the VM ability of tumour cells has since been proposed [57] based on the TFA developed for the assessment of angiogenesis by endothelial cells [58, 59]. During this assay endothelial cells migrate through a basement membrane matrix, such as Matrigel®, and align to form networks of tube-like structures resembling angiogenic vessels [59]. Acquisition of this endothelial phenotype by tumour cells during a TFA was the most common method used to identify both VM and tumour to endothelial transdifferentiation in vitro. Identification of markers expressed by tumour cells during or subsequent to tube formation was uncommon, though markers again included lack of CD31 in VM studies and expression of CD31 in transdifferentiation studies [30, 34]. Further distinctions between VM and endothelial transdifferentiation were made using additional techniques, such as PCR and Western blot, to indicate expression of markers of interest at baseline or under certain growth conditions, for example, growth of GSCs in EGM to promote endothelial differentiation prior to conducting a TFA. VM was distinguished from endothelial transdifferentiation again based on CD31 expression using these techniques. Other markers noted in studies that identified VM-capable glioblastoma cells were expression of VE-cadherin, MMP-2, MMP-9, and EphA2, all of which have previously been associated with VM in melanoma [5, 7, 10–12]. In addition to CD31, endothelial cells resulting from tumour cell transdifferentiation were reported to express various other markers that are generally considered to be vascular endothelial cell-specific, such as CD34, vWF, and VE-cadherin. Identification of VE-cadherin as a marker of both processes provides an example of the overlap in pathways, such as vascular signalling, that are associated with VM and endothelial transdifferentiation.

Uncertainty relating to which markers can be attributed to VM, endothelial transdifferentiation, or both, also arises from the inability to directly compare results between studies, which was evident in studies that performed TFAs. Despite nearly all studies reporting some form of tumour-derived vessel formation, these results were based on TFAs in which aspects such as growth conditions, incubation time, cell density, and cell type (i.e., commercially available cell lines, patient-derived tumour cells, or isolated GSCs) varied greatly, and in some cases these details were not specified. Some differences in the TFA occurred between VM and endothelial transdifferentiation groups, such as the use of commercially available cell lines in VM studies and the use of stem cell-only populations in transdifferentiation studies. However, one study reported tube formation ability and lack of CD31 and CD34 expression, and concluded that cells were undergoing VM, when the TFA was performed using GSCs isolated from patient tumours grown in EGM [16]. These aspects of the TFA method were common to transdifferentiation studies, in which presence of endothelial cell markers was frequently reported. Specific details of the TFA such as seeding density and time taken for tube formation to occur may differ for glioblastoma cells from what is recommended for endothelial cells, so determining optimal TFA conditions for the cells being used will be important in more fully characterising blood vessel formation ability of glioblastoma cells and providing insight into the conditions under which it occurs.

This systematic review provides an overview of the cellular and tissue characteristics that have so far been used to identify tumour blood vessels consisting of either VM or endothelial cells arising from tumour cell transdifferentiation in human glioblastoma. Assessing studies that use multiple methods allowed for variation in definitions and descriptions of tumour-derived vasculature in glioblastoma to be more fully explored. However, the inclusion of a wide range of study designs presents some limitations. For example, it precluded the use of a single quality assessment or risk of bias tool, and included study designs such as in vitro preclinical research, for which there are no standardised risk of bias tools. Quality of reporting within the included studies was therefore not fully determined. While direct comparisons between studies was also a challenge, sources of discrepancy have been noted for the most frequently used in vitro technique, the TFA. A more in-depth analysis of this subset of studies in the future would allow for the consistent design of reproducible experiments able to confirm the presence (or absence) of the markers reported here as being involved in the processes of VM and tumour to endothelial transdifferentiation.

## Conclusion

The results of this review suggest that expression of endothelial markers CD31 and/or CD34 distinguishes tumour to endothelial transdifferentiation from VM in glioblastoma. However, additional markers are required to confirm the tumour origin of potential glioblastoma-derived endothelial cells, and identification of tumour-specific markers for this purpose has not been consistent. Further research is also required to confirm the reliability of markers distinguishing VM from tumour to endothelial transdifferentiation in comparable studies.

## Electronic supplementary material

Below is the link to the electronic supplementary material.

Supplementary Material 1

Supplementary Material 2

## Data Availability

The datasets used and/or analysed during the current study are available from the corresponding author on reasonable request.

## Abbreviations

EGFR: epidermal growth factor receptor
EGM: endothelial cell growth medium
ELISA: enzyme-linked immunosorbent assay
FISH: fluorescence in situ hybridisation
GFAP: glial fibrillary acidic protein
GSC: glioblastoma stem-like cell
HMVEC: human microvascular endothelial cell
HUVEC: human umbilical vein endothelial cell
IF: immunofluorescence
IHC: immunohistochemistry
MMP: matrix metalloproteinase
PAS: periodic acid-Schiff
SMa: smooth muscle actin
TFA: tube formation assay
VEGF: vascular endothelial growth factor
VM: vasculogenic mimicry
vWF: von Willebrand factor

## References

[1] Chung AS, Lee J, Ferrara N (2010). Targeting the tumour vasculature: insights from physiological angiogenesis. Nat Rev Cancer.

[2] Carmeliet P, Jain RK (2011). Principles and mechanisms of vessel normalization for cancer and other angiogenic diseases. Nat Rev Drug Discov.

[3] Maniotis AJ, Folberg R, Hess A, Seftor EA, Gardner LMG, Pe’er J (1999). Vascular Channel formation by human melanoma cells in vivo and in Vitro: Vasculogenic Mimicry. Am J Pathol.

[4] Coultas L, Chawengsaksophak K, Rossant J (2005). Endothelial cells and VEGF in vascular development. Nature.

[5] Hendrix MJC, Seftor EA, Meltzer PS, Gardner LMG, Hess AR, Kirschmann DA (2001). Expression and functional significance of VE-cadherin in aggressive human melanoma cells: role in vasculogenic mimicry. Proc Natl Acad Sci.

[6] Hess AR, Margaryan NV, Seftor EA, Hendrix MJC (2007). Deciphering the signaling events that promote melanoma tumor cell vasculogenic mimicry and their link to embryonic vasculogenesis: role of the eph receptors. Dev Dyn.

[7] Seftor REB, Seftor EA, Koshikawa N, Meltzer PS, Gardner LMG, Bilban M (2001). Cooperative interactions of laminin 5 γ2 chain, Matrix Metalloproteinase-2, and membrane Type-1-Matrix/Metalloproteinase are required for mimicry of embryonic vasculogenesis by aggressive melanoma. Cancer Res.

[8] Hardy KM, Kirschmann DA, Seftor EA, Margaryan NV, Postovit L-M, Strizzi L (2010). Regulation of the embryonic morphogen nodal by Notch4 facilitates Manifestation of the aggressive Melanoma phenotype. Cancer Res.

[9] Topczewska JM, Postovit L-M, Margaryan NV, Sam A, Hess AR, Wheaton WW (2006). Embryonic and tumorigenic pathways converge via nodal signaling: role in melanoma aggressiveness. Nat Med.

[10] Hess AR, Seftor EA, Gruman LM, Kinch MS, Seftor REB, Hendrix MJC (2006). VE-cadherin regulates EphA2 in aggressive melanoma cells through a novel signaling pathway: implications for vasculogenic mimicry. Cancer Biol Ther.

[11] Hess AR, Seftor EA, Gardner LMG, Carles-Kinch K, Schneider GB, Seftor REB (2001). Molecular regulation of Tumor Cell Vasculogenic Mimicry by Tyrosine Phosphorylation: role of epithelial cell kinase (Eck/EphA2). Cancer Res.

[12] Hess AR, Seftor EA, Seftor REB, Hendrix MJC (2003). Phosphoinositide 3-Kinase regulates membrane type 1-Matrix metalloproteinase (MMP) and MMP-2 activity during Melanoma Cell Vasculogenic Mimicry. Cancer Res.

[13] Sun B, Zhang D, Zhang S, Zhang W, Guo H, Zhao X (2007). Hypoxia influences vasculogenic mimicry channel formation and tumor invasion-related protein expression in melanoma. Cancer Lett.

[14] Comito G, Calvani M, Giannoni E, Bianchini F, Calorini L, Torre E (2011). HIF-1α stabilization by mitochondrial ROS promotes Met-dependent invasive growth and vasculogenic mimicry in melanoma cells. Free Radic Biol Med.

[15] Yue W-Y, Chen Z-P (2005). Does Vasculogenic Mimicry Exist in Astrocytoma?. J Histochem Cytochem.

[16] El Hallani S, Boisselier B, Peglion F, Rousseau A, Colin C, Idbaih A (2010). A new alternative mechanism in glioblastoma vascularization: tubular vasculogenic mimicry. Brain.

[17] Liu X, Zhang Q, Mu Y, Zhang X, Sai K, Pang JC-S (2011). Clinical significance of vasculogenic mimicry in human gliomas. J Neurooncol.

[18] Louis DN, Perry A, Wesseling P, Brat DJ, Cree IA, Figarella-Branger D (2021). The 2021 WHO classification of tumors of the Central Nervous System: a summary. Neuro-Oncol.

[19] Australian Institute of Health and Welfare (2017). Brain and other central nervous system cancers.

[20] Stupp R, Weller M, Belanger K, Bogdahn U, Ludwin SK, Lacombe D (2005). Radiotherapy plus Concomitant and Adjuvant Temozolomide for Glioblastoma. N Engl J Med.

[21] Louis DN, Ohgaki H, Wiestler OD, Cavenee WK (2016). WHO classification of Tumours of the Central Nervous System. Revised 4th.

[22] Friedman HS, Prados MD, Wen PY, Mikkelsen T, Schiff D, Abrey LE (2009). Bevacizumab alone and in Combination with Irinotecan in Recurrent Glioblastoma. J Clin Oncol.

[23] Gilbert MR, Dignam JJ, Armstrong TS, Wefel JS, Blumenthal DT, Vogelbaum MA (2014). A Randomized Trial of Bevacizumab for newly diagnosed Glioblastoma. N Engl J Med.

[24] Wick W, Gorlia T, Bendszus M, Taphoorn M, Sahm F, Harting I (2017). Lomustine and Bevacizumab in Progressive Glioblastoma. N Engl J Med.

[25] Chinot OL, Wick W, Mason W, Henriksson R, Saran F, Nishikawa R (2014). Bevacizumab plus Radiotherapy–Temozolomide for newly diagnosed Glioblastoma. N Engl J Med.

[26] Wang R, Chadalavada K, Wilshire J, Kowalik U, Hovinga KE, Geber A (2010). Glioblastoma stem-like cells give rise to tumour endothelium. Nature.

[27] Ricci-Vitiani L, Pallini R, Biffoni M, Todaro M, Invernici G, Cenci T (2010). Tumour vascularization via endothelial differentiation of glioblastoma stem-like cells. Nature.

[28] Mao X-g, Xue X-y, Wang L, Zhang X, Yan M, Tu Y -y., editors. CDH5 is specifically activated in glioblastoma stemlike cells and contributes to vasculogenic mimicry induced by hypoxia. Neuro-Oncol. 2013;15:865–79. 10.1093/neuonc/not029 PMC3688011 23645533

[29] Porcù E, Maule F, Boso D, Rampazzo E, Barbieri V, Zuccolotto G (2018). BMP9 counteracts the tumorigenic and pro-angiogenic potential of glioblastoma. Cell Death Differ.

[30] Scully S, Francescone R, Faibish M, Bentley B, Taylor SL, Oh D (2012). Transdifferentiation of Glioblastoma Stem-Like cells into Mural cells drives vasculogenic mimicry in Glioblastomas. J Neurosci.

[31] Shaifer CA, Huang J, Lin PC (2010). Glioblastoma cells incorporate into tumor vasculature and contribute to vascular radioresistance. Int J Cancer.

[32] Zhu Y, Liu X, Zhao P, Zhao H, Gao W, Wang L. Celastrol Suppresses Glioma Vasculogenic Mimicry Formation and Angiogenesis by Blocking the PI3K/Akt/mTOR Signaling Pathway.Front Pharmacol. 2020;11. 10.3389/fphar.2020.00025 PMC7025498 32116702

[33] Fonsatti E, Altomonte M, Nicotra MR, Natali PG, Maio M (2003). Endoglin (CD105): a powerful therapeutic target on tumor-associated angiogenetic blood vessels. Oncogene.

[34] Bergès R, Tchoghandjian A, Sergé A, Honoré S, Figarella-Branger D, Bachmann F (2020). EB1-dependent long survival of glioblastoma-grafted mice with the oral tubulin-binder BAL101553 is associated with inhibition of tumor angiogenesis. Oncotarget.

[35] Deshors P, Toulas C, Arnauduc F, Malric L, Siegfried A, Nicaise Y (2019). Ionizing radiation induces endothelial transdifferentiation of glioblastoma stem-like cells through the Tie2 signaling pathway. Cell Death Dis.

[36] The Cancer Genome Atlas Research Network (2008). Comprehensive genomic characterization defines human glioblastoma genes and core pathways. Nature.

[37] Liu Z, Sun B, Qi L, Li H, Gao J, Leng X (2012). Zinc finger E-box binding homeobox 1 promotes vasculogenic mimicry in colorectal cancer through induction of epithelial-to-mesenchymal transition. Cancer Sci.

[38] Mitra D, Bhattacharyya S, Alam N, Sen S, Mitra S, Mandal S (2020). Phosphorylation of EphA2 receptor and vasculogenic mimicry is an indicator of poor prognosis in invasive carcinoma of the breast. Breast Cancer Res Treat.

[39] Sun B, Zhang S, Zhang D, Du J, Guo H, Zhao X (2006). Vasculogenic mimicry is associated with high tumor grade, invasion and metastasis, and short survival in patients with hepatocellular carcinoma. Oncol Rep.

[40] Williamson SC, Metcalf RL, Trapani F, Mohan S, Antonello J, Abbott B et al. Vasculogenic mimicry in small cell lung cancer.Nat Commun. 2016;7. 10.1038/ncomms13322 PMC5105195 27827359

[41] Wu Y, Du K, Guan W, Wu D, Tang H, Wang N (2020). A novel definition of microvessel density in renal cell carcinoma: angiogenesis plus vasculogenic mimicry. Oncol Lett.

[42] Mei X, Chen Y-S, Chen F-R, Xi S-Y, Chen Z-P (2017). Glioblastoma stem cell differentiation into endothelial cells evidenced through live-cell imaging. Neuro-Oncol.

[43] Zhao C, Gomez GA, Zhao Y, Yang Y, Cao D, Lu J (2018). ETV2 mediates endothelial transdifferentiation of glioblastoma. Signal Transduct Target Ther.

[44] He H, Niu CS, Li MW (2012). Correlation between glioblastoma stem-like cells and tumor vascularization. Oncol Rep.

[45] Dong J, Zhao Y, Huang Q, Fei X, Diao Y, Shen Y (2011). Glioma Stem/Progenitor cells contribute to Neovascularization via Transdifferentiation. Stem Cell Rev Rep.

[46] Calabrese C, Poppleton H, Kocak M, Hogg TL, Fuller C, Hamner B (2007). A Perivascular Niche for Brain Tumor Stem cells. Cancer Cell.

[47] Glumac PM, LeBeau AM. The role of CD133 in cancer: a concise review.Clin Transl Med. 2018;7. 10.1186/s40169-018-0198-1 PMC6035906 29984391

[48] Uchida N, Buck DW, He D, Reitsma MJ, Masek M, Phan TV (2000). Direct isolation of human central nervous system stem cells. Proc Natl Acad Sci.

[49] Singh SK, Hawkins C, Clarke ID, Squire JA, Bayani J, Hide T (2004). Identification of human brain tumour initiating cells. Nature.

[50] Akino T, Hida K, Hida Y, Tsuchiya K, Freedman D, Muraki C (2009). Cytogenetic abnormalities of Tumor-Associated endothelial cells in human malignant tumors. Am J Pathol.

[51] Hida K, Hida Y, Amin DN, Flint AF, Panigrahy D, Morton CC (2004). Tumor-Associated endothelial cells with cytogenetic abnormalities. Cancer Res.

[52] Kondoh M, Ohga N, Akiyama K, Hida Y, Maishi N, Towfik AM (2013). Hypoxia-Induced reactive oxygen species cause chromosomal abnormalities in endothelial cells in the Tumor Microenvironment. PLoS ONE.

[53] Lee J, Kotliarova S, Kotliarov Y, Li A, Su Q, Donin NM (2006). Tumor stem cells derived from glioblastomas cultured in bFGF and EGF more closely mirror the phenotype and genotype of primary tumors than do serum-cultured cell lines. Cancer Cell.

[54] Pollard SM, Yoshikawa K, Clarke ID, Danovi D, Stricker S, Russell R (2009). Glioma Stem Cell Lines expanded in Adherent Culture have tumor-specific phenotypes and are suitable for Chemical and genetic screens. Cell Stem Cell.

[55] Haddad AF, Young JS, Amara D, Berger MS, Raleigh DR, Aghi MK (2021). Mouse models of glioblastoma for the evaluation of novel therapeutic strategies. Neuro-Oncol Adv.

[56] Qiang L, Yang Y, Ma Y-J, Chen F-H, Zhang L-B, Liu W (2009). Isolation and characterization of cancer stem like cells in human glioblastoma cell lines. Cancer Lett.

[57] Francescone RA III, Faibish M, Shao R. A Matrigel-Based Tube Formation Assay to Assess the Vasculogenic Activity of Tumor Cells.J Vis Exp. 2011;:3040. 10.3791/3040 PMC3230200 21931289

[58] Kubota Y, Kleinman HK, Martin GR, Lawley TJ (1988). Role of laminin and basement membrane in the morphological differentiation of human endothelial cells into capillary-like structures. J Cell Biol.

[59] Arnaoutova I, Kleinman HK (2010). In vitro angiogenesis: endothelial cell tube formation on gelled basement membrane extract. Nat Protoc.

