# Supplementary material for: Characteristics of vasculogenic mimicry and tumour to endothelial transdifferentiation in human glioblastoma: a systematic review
Source: BMC Cancer. 2023 Feb 23;23:185. doi: 10.1186/s12885-023-10659-y (PMC9948311; doi:10.1186/s12885-023-10659-y)
Supplement: Supplementary file 1 — Supplementary Material 1 [file 12885_2023_10659_MOESM1_ESM.docx]

**Supplementary references**

1. Alowaidi F, Hashimi SM, Nguyen M, Meshram M, Alqurashi N, Cavanagh BL, et al. Investigating the role of CRIPTO‐1 (TDGF‐1) in glioblastoma multiforme U87 cell line. J of Cellular Biochemistry. 2019;120:7412–27.

2. Angara K, Borin TF, Rashid MH, Lebedyeva I, Ara R, Lin P-C, et al. CXCR2-Expressing Tumor Cells Drive Vascular Mimicry in Antiangiogenic Therapy–Resistant Glioblastoma. Neoplasia. 2018;20:1070–82.

3. Angara K, Rashid MH, Shankar A, Ara R, Iskander A, Borin TF, et al. Vascular mimicry in glioblastoma following anti-angiogenic and anti-20-HETE therapies. Histology and histopathology. 2017;32:917–28.

4. Baisiwala S, Auffinger B, Caragher SP, Shireman JM, Ahsan R, Lee G, et al. Chemotherapeutic Stress Induces Transdifferentiation of Glioblastoma Cells to Endothelial Cells and Promotes Vascular Mimicry. Stem Cells International. 2019;2019:1–14.

5. Bergès R, Tchoghandjian A, Sergé A, Honoré S, Figarella-Branger D, Bachmann F, et al. EB1-dependent long survival of glioblastoma-grafted mice with the oral tubulin-binder BAL101553 is associated with inhibition of tumor angiogenesis. Oncotarget. 2020;11:759–74.

6. Bieche I, Vacher S, Vallerand D, Richon S, Hatem R, De Plater L, et al. Vasculature analysis of patient derived tumor xenografts using species-specific PCR assays: evidence of tumor endothelial cells and atypical VEGFA-VEGFR1/2 signalings. BMC Cancer. 2014;14:178.

7. Bougnaud S, Golebiewska A, Oudin A, Keunen O, Harter PN, Mäder L, et al. Molecular crosstalk between tumour and brain parenchyma instructs histopathological features in glioblastoma. Oncotarget. 2016;7:31955–71.

8. Cai H, Wang J, Xi S, Ni X, Chen Y, Yu Y, et al. Tenascin-c mediated vasculogenic mimicry formation via regulation of MMP2/MMP9 in glioma. Cell Death Dis. 2019;10:879.

9. Cao W, Xu C, Li X, Yang X. Twist1 promotes astrocytoma development by stimulating vasculogenic mimicry. Oncol Lett. 2019;18:846–55.

10. Carlson JC, Cantu Gutierrez M, Lozzi B, Huang-Hobbs E, Turner WD, Tepe B, et al. Identification of diverse tumor endothelial cell populations in malignant glioma. Neuro-Oncology. 2021;23:932–44.

11. Chen L, Lin Z-X, Lin G-S, Zhou C-F, Chen Y-P, Wang X-F, et al. Classification of microvascular patterns via cluster analysis reveals their prognostic significance in glioblastoma. Human Pathology. 2015;46:120–8.

12. Chen Y, Jing Z, Luo C, Zhuang M, Xia J, Chen Z, et al. Vasculogenic mimicry–potential target for glioblastoma therapy: an in vitro and in vivo study. Med Oncol. 2012;29:324–31.

13. Chiao M-T, Yang Y-C, Cheng W-Y, Shen C-C, Ko J-L. CD133+ Glioblastoma Stem-Like Cells Induce Vascular Mimicry in Vivo. CNR. 2011;8:210–9.

14. Chiba R, Akiya M, Hashimura M, Oguri Y, Inukai M, Hara A, et al. ALK signaling cascade confers multiple advantages to glioblastoma cells through neovascularization and cell proliferation. PLoS ONE. 2017;12:e0183516.

15. Choi EJ, Cho BJ, Lee DJ, Hwang YH, Chun SH, Kim HH, et al. Enhanced cytotoxic effect of radiation and temozolomide in malignant glioma cells: targeting PI3K-AKT-mTOR signaling, HSP90 and histone deacetylases. BMC Cancer. 2014;14:17.

16. Costa H, Xu X, Overbeek G, Vasaikar S, Patro CPK, Kostopoulou ON, et al. *Human cytomegalovirus* may promote tumour progression by upregulating arginase-2. Oncotarget. 2016;7:47221–31.

17. Cui C, Chen X, Liu Y, Cao B, Xing Y, Liu C, et al. β1,4-Galactosyltransferase V activates Notch1 signaling in glioma stem-like cells and promotes their transdifferentiation into endothelial cells. Journal of Biological Chemistry. 2018;293:2219–30.

18. De Pascalis I, Morgante L, Pacioni S, D’Alessandris QG, Giannetti S, Martini M, et al. Endothelial trans-differentiation in glioblastoma recurring after radiotherapy. Mod Pathol. 2018;31:1361–6.

19. Deshors P, Toulas C, Arnauduc F, Malric L, Siegfried A, Nicaise Y, et al. Ionizing radiation induces endothelial transdifferentiation of glioblastoma stem-like cells through the Tie2 signaling pathway. Cell Death Dis. 2019;10:816.

20. Dong J, Zhang Q, Huang Q, Chen H, Shen Y, Fei X, et al. Glioma stem cells involved in tumor tissue remodeling in a xenograft model: Laboratory investigation. JNS. 2010;113:249–60.

21. Dong J, Zhao Y, Huang Q, Fei X, Diao Y, Shen Y, et al. Glioma Stem/Progenitor Cells Contribute to Neovascularization via Transdifferentiation. Stem Cell Rev and Rep. 2011;7:141–52.

22. Duan S. Silencing the autophagy-specific gene Beclin-1 contributes to attenuated hypoxia-induced vasculogenic mimicry formation in glioma. CBM. 2018;21:565–74.

23. El Hallani S, Boisselier B, Peglion F, Rousseau A, Colin C, Idbaih A, et al. A new alternative mechanism in glioblastoma vascularization: tubular vasculogenic mimicry. Brain. 2010;133:973–82.

24. El Hallani S, Colin C, El Houfi Y, Idbaih A, Boisselier B, Marie Y, et al. Tumor and Endothelial Cell Hybrids Participate in Glioblastoma Vasculature. BioMed Research International. 2014;2014:1–9.

25. Eom K-Y, Cho BJ, Choi EJ, Kim J-H, Chie EK, Wu H-G, et al. The Effect of Chemoradiotherapy with SRC Tyrosine Kinase Inhibitor, PP2 and Temozolomide on Malignant Glioma Cells *In Vitro* and *In Vivo*. Cancer Res Treat. 2016;48:687–97.

26. Feng X, Yao J, Gao X, Jing Y, Kang T, Jiang D, et al. Multi-targeting Peptide-Functionalized Nanoparticles Recognized Vasculogenic Mimicry, Tumor Neovasculature, and Glioma Cells for Enhanced Anti-glioma Therapy. ACS Appl Mater Interfaces. 2015;7:27885–99.

27. Francescone III RA, Faibish M, Shao R. A Matrigel-Based Tube Formation Assay to Assess the Vasculogenic Activity of Tumor Cells. JoVE. 2011;:3040.

28. Francescone R, Scully S, Bentley B, Yan W, Taylor SL, Oh D, et al. Glioblastoma-derived Tumor Cells Induce Vasculogenic Mimicry through Flk-1 Protein Activation. Journal of Biological Chemistry. 2012;287:24821–31.

29. Gao Y, Yu H, Liu Y, Liu X, Zheng J, Ma J, et al. Long Non-Coding RNA HOXA-AS2 Regulates Malignant Glioma Behaviors and Vasculogenic Mimicry Formation via the MiR-373/EGFR Axis. Cell Physiol Biochem. 2018;45:131–47.

30. Gravina GL, Mancini A, Colapietro A, Delle Monache S, Sferra R, Pompili S, et al. The Brain Penetrating and Dual TORC1/TORC2 Inhibitor, RES529, Elicits Anti-Glioma Activity and Enhances the Therapeutic Effects of Anti-Angiogenetic Compounds in Preclinical Murine Models. Cancers. 2019;11:1604.

31. Greish K, Jasim A, Parayath N, Abdelghany S, Alkhateeb A, Taurin S, et al. Micellar formulations of Crizotinib and Dasatinib in the management of glioblastoma multiforme. Journal of Drug Targeting. 2018;26:692–708.

32. Guerra-Rebollo M, Garrido C, Sánchez-Cid L, Soler-Botija C, Meca-Cortés O, Rubio N, et al. Targeting of replicating CD133 and OCT4/SOX2 expressing glioma stem cells selects a cell population that reinitiates tumors upon release of therapeutic pressure. Sci Rep. 2019;9:9549.

33. Guichet P-O, Guelfi S, Teigell M, Hoppe L, Bakalara N, Bauchet L, et al. Notch1 Stimulation Induces a Vascularization Switch With Pericyte-Like Cell Differentiation of Glioblastoma Stem Cells. Stem Cells. 2015;33:21–34.

34. Guo J, Cai H, Liu X, Zheng J, Liu Y, Gong W, et al. Long Non-coding RNA LINC00339 Stimulates Glioma Vasculogenic Mimicry Formation by Regulating the miR-539-5p/TWIST1/MMPs Axis. Molecular Therapy - Nucleic Acids. 2018;10:170–86.

35. Guo X, Xu S, Gao X, Wang J, Xue H, Chen Z, et al. Macrophage migration inhibitory factor promotes vasculogenic mimicry formation induced by hypoxia via CXCR4/AKT/EMT pathway in human glioblastoma cells. Oncotarget. 2017;8:80358–72.

36. Han G, Li Y, Cao Y, Yue Z, Zhang Y, Wang L, et al. Overexpression of leptin receptor in human glioblastoma: Correlation with vasculogenic mimicry and poor prognosis. Oncotarget. 2017;8:58163–71.

37. He H, Niu CS, Li MW. Correlation between glioblastoma stem-like cells and tumor vascularization. Oncol Rep. 2012;27:45–50.

38. He X, Liu J, Wang X, Liu T, Yang L, Li C, et al. The embryonic stem cell microenvironment inhibits mouse glioma cell proliferation by regulating the PI3K/AKT pathway. Transl Cancer Res TCR. 2021;10:487–98.

39. Huang D, Zhang S, Zhong T, Ren W, Yao X, Guo Y, et al. Multi-targeting NGR-modified liposomes recognizing glioma tumor cells and vasculogenic mimicry for improving anti-glioma therapy. Oncotarget. 2016;7:43616–28.

40. Huang M, Ke Y, Sun X, Yu L, Yang Z, Zhang Y, et al. Mammalian target of rapamycin signaling is involved in the vasculogenic mimicry of glioma via hypoxia-inducible factor-1α. Oncology Reports. 2014;32:1973–80.

41. Jin Z, Zhan T, Tao J, Xu B, Zheng H, Cheng Y, et al. *MicroRNA* - *34a* induces transdifferentiation of glioma stem cells into vascular endothelial cells by targeting Notch pathway. Bioscience, Biotechnology, and Biochemistry. 2017;81:1899–907.

42. Jing F, Ruan X, Liu X, Yang C, Wang D, Zheng J, et al. The PABPC5/HCG15/ZNF331 Feedback Loop Regulates Vasculogenic Mimicry of Glioma via STAU1-Mediated mRNA Decay. Molecular Therapy - Oncolytics. 2020;17:216–31.

43. Ke C, Luo J, Cen Z, Li Y, Cai H, Wang J, et al. Dual antivascular function of human fibulin‐3 variant, a potential new drug discovery strategy for glioblastoma. Cancer Sci. 2020;111:940–50.

44. Kong Y, Feng Z-C, Zhang Y-L, Liu X-F, Ma Y, Zhao Z-M, et al. Identification of Immune-Related Genes Contributing to the Development of Glioblastoma Using Weighted Gene Co-expression Network Analysis. Front Immunol. 2020;11:1281.

45. Kuang X-Y, Ren Y, Chen C, Su J, Li H-M, Liu S-J, et al. Quantitative analysis for the differences in vasculogenic activity and sensitivity to angiogenic stimulants between human glioma cells and normal endothelial cells. Brain Research. 2020;1748:147082.

46. Li C, Chen Y, Zhang Q, Guo C, Chen F, Xi S, et al. Expression of Twist associated to microcirculation patterns of human glioma correlated with progression and survival of the patient. In: International Review of Neurobiology. Elsevier; 2020. p. 201–17.

47. Li G, Huang M, Cai Y, Ke Y, Yang Y, Sun X. miR‑141 inhibits glioma vasculogenic mimicry by controlling EphA2 expression. Mol Med Report. 2018;18:1395–404.

48. Li J, Ke Y, Huang M, Huang S, Liang Y. Inhibitory effects of B-cell lymphoma 2 on the vasculogenic mimicry of hypoxic human glioma cells. Experimental and Therapeutic Medicine. 2015;9:977–81.

49. Li X, Xue Y, Liu X, Zheng J, Shen S, Yang C, et al. ZRANB2/SNHG20/FOXK1 Axis regulates Vasculogenic mimicry formation in glioma. J Exp Clin Cancer Res. 2019;38:68.

50. Li X, Shi L, Li Y, Li Q, Duan X, Wang Y, et al. The enhanced treatment efficacy of invasive brain glioma by dual-targeted artemether plus paclitaxel micelles. Artificial Cells, Nanomedicine, and Biotechnology. 2020;48:983–96.

51. Liang C, Shangguan J, Yang L, Guo S. Downregulation of astrocyte elevated gene‑1 expression inhibits the development of vasculogenic mimicry in gliomas. Exp Ther Med. 2020;21:1–1.

52. Liang Y, Huang M, Li J, Sun X, Jiang X, Li L, et al. Curcumin inhibits vasculogenic mimicry through the downregulation of erythropoietin-producing hepatocellular carcinoma-A2, phosphoinositide 3-kinase and matrix metalloproteinase-2. Oncology Letters. 2014;8:1849–55.

53. Ling G-Q, Liu Y-J, Ke Y-Q, Chen L, Jiang X-D, Jiang C-L, et al. All-trans retinoic acid impairs the vasculogenic mimicry formation ability of U87 stem-like cells through promoting differentiation. Molecular Medicine Reports. 2015;12:165–72.

54. Ling G, Wang S, Song Z, Sun X, Liu Y, Jiang X, et al. Transforming growth factor-β is required for vasculogenic mimicry formation in glioma cell line U251MG. Cancer Biology & Therapy. 2011;12:978–88.

55. Liu X, Wang J, Li S, Li L, Huang M, Zhang Y, et al. Histone deacetylase 3 expression correlates with vasculogenic mimicry through the phosphoinositide3‐kinase / ERK– MMP –laminin5γ2 signaling pathway. Cancer Sci. 2015;106:857–66.

56. Liu X, Zhang Q, Mu Y, Zhang X, Sai K, Pang JC-S, et al. Clinical significance of vasculogenic mimicry in human gliomas. J Neurooncol. 2011;105:173–9.

57. Liu X, Wang X, Du W, Chen L, Wang G, Cui Y, et al. Suppressor of fused (Sufu) represses Gli1 transcription and nuclear accumulation, inhibits glioma cell proliferation, invasion and vasculogenic mimicry, improving glioma chemo-sensitivity and prognosis. Oncotarget. 2014;5:11681–94.

58. Liu Y, Li F, Yang YT, Xu XD, Chen JS, Chen TL, et al. IGFBP2 promotes vasculogenic mimicry formation via regulating CD144 and MMP2 expression in glioma. Oncogene. 2019;38:1815–31.

59. Liu Z, Li Y, Zhao W, Ma Y, Yang X. Demonstration of vasculogenic mimicry in astrocytomas and effects of Endostar on U251 cells. Pathology - Research and Practice. 2011;207:645–51.

60. Lu W-L, Ju R-J, Zeng F, Liu L, Mu L-M, Xie H-J, et al. Destruction of vasculogenic mimicry channels by targeting epirubicin plus celecoxib liposomes in treatment of brain glioma. IJN. 2016;:1131.

61. Mao X -g., Xue X -y., Wang L, Zhang X, Yan M, Tu Y -y., et al. CDH5 is specifically activated in glioblastoma stemlike cells and contributes to vasculogenic mimicry induced by hypoxia. Neuro-Oncology. 2013;15:865–79.

62. Mei X, Chen Y-S, Chen F-R, Xi S-Y, Chen Z-P. Glioblastoma stem cell differentiation into endothelial cells evidenced through live-cell imaging. Neuro-Oncology. 2017;19:1109–18.

63. Mei X, Chen Y, Zhang Q, Chen F, Xi S, Long Y, et al. Association between glioblastoma cell‐derived vessels and poor prognosis of the patients. Cancer Communications. 2020;40:211–21.

64. Pan Z, Zhu Q, You W, Shen C, Hu W, Chen X. Silencing of Mig-7 expression inhibits in-vitro invasiveness and vasculogenic mimicry of human glioma U87 Cells. NeuroReport. 2019;30:1135–42.

65. Pastorino O, Gentile MT, Mancini A, Del Gaudio N, Di Costanzo A, Bajetto A, et al. Histone Deacetylase Inhibitors Impair Vasculogenic Mimicry from Glioblastoma Cells. Cancers. 2019;11:747.

66. Porcù E, Maule F, Boso D, Rampazzo E, Barbieri V, Zuccolotto G, et al. BMP9 counteracts the tumorigenic and pro-angiogenic potential of glioblastoma. Cell Death Differ. 2018;25:1808–22.

67. Qiu X, Chen L, Wang C, Lin Z, Chen B, You N, et al. The Vascular Notch Ligands Delta-Like Ligand 4 (DLL4) and Jagged1 (JAG1) Have Opposing Correlations with Microvascularization but a Uniform Prognostic Effect in Primary Glioblastoma: A Preliminary Study. World Neurosurgery. 2016;88:447–58.

68. Qu M, Yu J, Liu H, Ren Y, Ma C, Bu X, et al. The Candidate Tumor Suppressor Gene SLC8A2 Inhibits Invasion, Angiogenesis and Growth of Glioblastoma. Mol Cells. 2017;40:761–72.

69. Qu Y, Zhang L, Rong Z, He T, Zhang S. Number of glioma polyploid giant cancer cells (PGCCs) associated with vasculogenic mimicry formation and tumor grade in human glioma. J Exp Clin Cancer Res. 2013;32:75.

70. Ricci-Vitiani L, Pallini R, Biffoni M, Todaro M, Invernici G, Cenci T, et al. Tumour vascularization via endothelial differentiation of glioblastoma stem-like cells. Nature. 2010;468:824–8.

71. Rocha R, Torres Á, Ojeda K, Uribe D, Rocha D, Erices J, et al. The Adenosine A3 Receptor Regulates Differentiation of Glioblastoma Stem-Like Cells to Endothelial Cells under Hypoxia. IJMS. 2018;19:1228.

72. Rong X, Huang B, Qiu S, Li X, He L, Peng Y. Tumor-associated macrophages induce vasculogenic mimicry of glioblastoma multiforme through cyclooxygenase-2 activation. Oncotarget. 2016;7:83976–86.

73. Ruan H, Chai Z, Shen Q, Chen X, Su B, Xie C, et al. A novel peptide ligand RAP12 of LRP1 for glioma targeted drug delivery. Journal of Controlled Release. 2018;279:306–15.

74. Schmitt F, Kasparkova J, Brabec V, Begemann G, Schobert R, Biersack B. New (arene)ruthenium(II) complexes of 4‑aryl‑4H‑naphthopyrans with anticancer and anti-vascular activities. Journal of Inorganic Biochemistry. 2018;184:69–78.

75. Scully S, Francescone R, Faibish M, Bentley B, Taylor SL, Oh D, et al. Transdifferentiation of Glioblastoma Stem-Like Cells into Mural Cells Drives Vasculogenic Mimicry in Glioblastomas. Journal of Neuroscience. 2012;32:12950–60.

76. Serrano-Garrido O, Peris-Torres C, Redondo-García S, Asenjo HG, Plaza-Calonge M del C, Fernandez-Luna JL, et al. ADAMTS1 Supports Endothelial Plasticity of Glioblastoma Cells with Relevance for Glioma Progression. Biomolecules. 2020;11:44.

77. Shaifer CA, Huang J, Lin PC. Glioblastoma cells incorporate into tumor vasculature and contribute to vascular radioresistance. Int J Cancer. 2010;127:2063–75.

78. Sharma I, Singh A, Siraj F, Saxena S. IL-8/CXCR1/2 signalling promotes tumor cell proliferation, invasion and vascular mimicry in glioblastoma. J Biomed Sci. 2018;25:62.

79. Smith SJ, Ward JH, Tan C, Grundy RG, Rahman R. dimensional culture identifies a role for VEGF and FGFR in a. :15.

80. Soda Y, Marumoto T, Friedmann-Morvinski D, Soda M, Liu F, Michiue H, et al. Transdifferentiation of glioblastoma cells into vascular endothelial cells. Proc Natl Acad Sci USA. 2011;108:4274–80.

81. Song Y, Mu L, Han X, Li Q, Dong B, Li H, et al. MicroRNA-9 inhibits vasculogenic mimicry of glioma cell lines by suppressing Stathmin expression. J Neurooncol. 2013;115:381–90.

82. Song Y, Mu L, Han X, Liu X, Fu S. siRNA targeting stathmin inhibits invasion and enhances chemotherapy sensitivity of stem cells derived from glioma cell lines. ABBS. 2014;46:1034–40.

83. Sun Q, Mu L, Qiao W, Li H, Tang J, Wang C, et al. Inhibition of SHP-2 promotes radiosensitivity in glioma. Molecular Medicine Reports. 2015;12:3563–8.

84. Tian Y, Huang Y, Gao P, Chen T. Nucleus-targeted DNA tetrahedron as a nanocarrier of metal complexes for enhanced glioma therapy. Chem Commun. 2018;54:9394–7.

85. Trisciuoglio D, Tupone MG, Desideri M, Di Martile M, Gabellini C, Buglioni S, et al. BCL-XL overexpression promotes tumor progression-associated properties. Cell Death Dis. 2017;8:3216.

86. Wang D, Ruan X, Liu X, Xue Y, Shao L, Yang C, et al. SUMOylation of PUM2 promotes the vasculogenic mimicry of glioma cells via regulating CEBPD. Clinical and Translational Medicine. 2020;10.

87. Wang H, Fu J, Xu D, Xu W, Wang S, Zhang L, et al. Downregulation of Pygopus 2 inhibits vascular mimicry in glioma U251 cells by suppressing the canonical Wnt signaling pathway. Oncology Letters. 2016;11:678–84.

88. Wang M, Yang C, Liu X, Zheng J, Xue Y, Ruan X, et al. An upstream open reading frame regulates vasculogenic mimicry of glioma via ZNRD1‐AS1/miR‐499a‐5p/ELF1/EMI1 pathway. J Cellular Molecular Medi. 2020;24:6120–36.

89. Wang R, Chadalavada K, Wilshire J, Kowalik U, Hovinga KE, Geber A, et al. Glioblastoma stem-like cells give rise to tumour endothelium. Nature. 2010;468:829–33.

90. Wang S, Ke Y, Lu G, Song Z, Yu L, Xiao S, et al. Vasculogenic mimicry is a prognostic factor for postoperative survival in patients with glioblastoma. J Neurooncol. 2013;112:339–45.

91. Wang X, Li X, Ding J, Long X, Zhang H, Zhang X, et al. 3D bioprinted glioma microenvironment for glioma vascularization. J Biomed Mater Res. 2021;109:915–25.

92. Wu H-B, Yang S, Weng H-Y, Chen Q, Zhao X-L, Fu W-J, et al. Autophagy-induced KDR/VEGFR-2 activation promotes the formation of vasculogenic mimicry by glioma stem cells. Autophagy. 2017;13:1528–42.

93. Wu N, Zhao X, Liu M, Liu H, Yao W, Zhang Y, et al. Role of MicroRNA-26b in Glioma Development and Its Mediated Regulation on EphA2. PLoS ONE. 2011;6:e16264.

94. Xu S, Zhang J, Xue H, Guo X, Han X, Li T, et al. MicroRNA-584-3p reduces the vasculogenic mimicry of human glioma cells by regulating hypoxia-induced ROCK1 dependent stress fiber formation. neo. 2017;64:13–21.

95. Xue H, Gao X, Xu S, Zhang J, Guo X, Yan S, et al. MicroRNA-Let-7f reduces the vasculogenic mimicry of human glioma cells by regulating periostin-dependent migration. Oncology Reports. 2016;35:1771–7.

96. Xue W, Du X, Wu H, Liu H, Xie T, Tong H, et al. Aberrant glioblastoma neovascularization patterns and their correlation with DCE-MRI-derived parameters following temozolomide and bevacizumab treatment. Sci Rep. 2017;7:13894.

97. Yang W, Tan Z, Dong D, Ding Y, Meng H, Zhao Y, et al. Association of aquaporin‑1 with tumor migration, invasion and vasculogenic mimicry in glioblastoma multiforme. Mol Med Report. 2017;17:3206–11.

98. Yao X, Ping Y, Liu Y, Chen K, Yoshimura T, Liu M, et al. Vascular Endothelial Growth Factor Receptor 2 (VEGFR-2) Plays a Key Role in Vasculogenic Mimicry Formation, Neovascularization and Tumor Initiation by Glioma Stem-like Cells. PLoS ONE. 2013;8:e57188.

99. Yin H, Shao Y, Chen X. The effects of CD147 on the cell proliferation, apoptosis, invasion, and angiogenesis in glioma. Neurol Sci. 2017;38:129–36.

100. Yin T, Wu J, Hu Y, Zhang M, He J. Long non‐coding RNA HULC stimulates the epithelial–mesenchymal transition process and vasculogenic mimicry in human glioblastoma. Cancer Medicine. 2021;10:5270–82.

101. Ying M, Shen Q, Zhan C, Wei X, Gao J, Xie C, et al. A stabilized peptide ligand for multifunctional glioma targeted drug delivery. Journal of Controlled Release. 2016;243:86–98.

102. Ying M, Zhan C, Wang S, Yao B, Hu X, Song X, et al. Liposome-Based Systemic Glioma-Targeted Drug Delivery Enabled by All- d Peptides. ACS Appl Mater Interfaces. 2016;8:29977–85.

103. Yu S, Ruan X, Liu X, Zhang F, Wang D, Liu Y, et al. HNRNPD interacts with ZHX2 regulating the vasculogenic mimicry formation of glioma cells via linc00707/miR-651-3p/SP2 axis. Cell Death Dis. 2021;12:153.

104. Yue W-Y, Chen Z-P. Does Vasculogenic Mimicry Exist in Astrocytoma? J Histochem Cytochem. 2005;53:997–1002.

105. Zhang C, Chen W, Zhang X, Huang B, Chen A, He Y, et al. Galunisertib inhibits glioma vasculogenic mimicry formation induced by astrocytes. Sci Rep. 2016;6:23056.

106. Zhang L, Xu Y, Sun J, Chen W, Zhao L, Ma C, et al. M2-like tumor-associated macrophages drive vasculogenic mimicry through amplification of IL-6 expression in glioma cells. Oncotarget. 2017;8:819–32.

107. Zhang Z, Guo X, Guo X, Yu R, Qian M, Wang S, et al. MicroRNA-29a-3p delivery via exosomes derived from engineered human mesenchymal stem cells exerts tumour suppressive effects by inhibiting migration and vasculogenic mimicry in glioma. Aging. 2021;13:5055–68.

108. Zhao C, Gomez GA, Zhao Y, Yang Y, Cao D, Lu J, et al. ETV2 mediates endothelial transdifferentiation of glioblastoma. Sig Transduct Target Ther. 2018;3:4.

109. Zheng P-P, van der Weiden M, van der Spek PJ, Vincent AJPE, Kros JM. Intratumoral, not circulating, endothelial progenitor cells share genetic aberrations with glial tumor cells. J Cell Physiol. 2013;228:1383–90.

110. Zhou Y, Jin G, Mi R, Zhang J, Zhang J, Xu H, et al. Knockdown of P4HA1 inhibits neovascularization via targeting glioma stem cell-endothelial cell transdifferentiation and disrupting vascular basement membrane. Oncotarget. 2017;8:35877–89.

111. Zhu H, Dai C, He L, Xu A, Chen T. Iron (II) Polypyridyl Complexes as Antiglioblastoma Agents to Overcome the Blood-Brain Barrier and Inhibit Cell Proliferation by Regulating p53 and 4E-BP1 Pathways. Front Pharmacol. 2019;10:946.

112. Zhu Y, Liu X, Zhao P, Zhao H, Gao W, Wang L. Celastrol Suppresses Glioma Vasculogenic Mimicry Formation and Angiogenesis by Blocking the PI3K/Akt/mTOR Signaling Pathway. Front Pharmacol. 2020;11:25.

113. Zhu Y, Liu A, Zhang X, Qi L, Zhang L, Xue J, et al. The effect of benzyl isothiocyanate and its computer-aided design derivants targeting alkylglycerone phosphate synthase on the inhibition of human glioma U87MG cell line. Tumor Biol. 2015;36:3499–509.
